# Mitochondrial Haplogroup and the Risk of Acute Kidney Injury Following Cardiac Bypass Surgery

**DOI:** 10.1038/s41598-018-37944-2

**Published:** 2019-02-19

**Authors:** Nigel S. Kanagasundaram, Simon V. Baudouin, Sarah Rowling, Mahesh Prabhu, John H. Dark, Timothy H. J. Goodship, Patrick F. Chinnery, Gavin Hudson

**Affiliations:** 1 Renal Services, Newcastle upon Tyne Hospitals NHS Foundation Trust, Newcastle upon Tyne, NE7 7DN UK; 2 Institute of Cellular Medicine, Newcastle University, Newcastle upon Tyne, NE2 4HH UK; 3 Department of Anaesthesia, Newcastle upon Tyne Hospitals NHS Foundation Trust, Newcastle upon Tyne, NE1 4LP UK; 4 Cardiothoracic Services, Newcastle upon Tyne Hospitals NHS Foundation Trust, Newcastle upon Tyne, NE7 7DN UK; 5 Institute of Genetic Medicine, Newcastle University, Newcastle upon Tyne, NE1 3BZ UK; 6 MRC Mitochondrial Biology Unit, University of Cambridge, Cambridge, CB2 0XY UK

## Abstract

Although mitochondrial dysfunction plays a key role in the pathophysiology of acute kidney injury (AKI), the influence of mitochondrial genetic variability in this process remains unclear. We explored the association between the risk of post-cardiac bypass AKI and mitochondrial haplotype – inherited mitochondrial genomic variations of potentially functional significance. Our single-centre study recruited consecutive patients prior to surgery. Exclusions included stage 5 CKD, non-Caucasian race and subsequent off-pump surgery. Haplogroup analysis allowed characterisation of the study population using the common mutations and by phylogenetic supergroup (WXI and HV). Chi-square tests for association allowed the identification of potential predictors of AKI for use in logistic regression analysis. AKI occurred in 12.8% of the study population (n = 881; male 69.6%, non-diabetic 78.5%, median (interquartile range) age 68.0 (61.0–75.0) years). The haplogroup profile comprised H (42.7%), J (12.1%), T (10.9%), U (14.4%) and K (7.6%). Although the regression model was statistically significant (χ^2^ = 95.483, p < 0.0005), neither the phylogenetic supergroups nor any individual haplogroup was a significant contributor. We found no significant association between common European haplogroups and the risk of post-cardiac bypass AKI. However, given the major role of mitochondrial dysfunction in AKI, there is a need to replicate our findings in other cohorts and with other aetiologies of AKI.

## Introduction

Acute kidney injury (AKI) is a common adverse outcome of cardiac surgery and affects around 18% of patients^1^. The incidence of more severe AKI, requiring renal support, has been reported at 1–2% across various series^2–5^ and is associated with mortality rates of at least 45%^2,4,5^.

The pathogenesis of the AKI occurring after cardio-pulmonary bypass may include ischaemia-reperfusion injury, inflammation induced in the extracorporeal blood circuit, haemolysis, embolization and medication-related injury^1^. Disruption of cellular energetics appears to be a common pathway for many of these factors, as it is in all forms of ischaemic AKI^6^, with mitochondria playing a pivotal role in this process^7^. Subtle differences in mitochondrial function may, therefore, affect susceptibility to AKI through their impact on cellular energy production, free radical generation and metabolic uncoupling.

With this in mind, inherited variation in the mitochondrial genome has not only allowed human populations to be categorized into different mitochondrial haplogroups, but may also be of functional significance^8^; associations have been found, for instance, between mitochondrial haplotype and Alzheimer’s disease^9^, Parkinson’s disease^10^ and ischaemic stroke^11^, and with physiological functions such as sperm motility^12^. Work undertaken within our group has also shown that haplogroup H – the commonest haplotype in European populations^12^ - was associated with improved survival after sepsis in comparison to non-haplogroup H subjects^13^. This observation has physiological credibility as subjects with this haplotype have higher respiratory chain activity^12^ and so may be less prone to the risks of the tissue dysoxia that occurs in sepsis^14^.

A relationship between mitochondrial genetic variability, mitochondrial function and susceptibility to AKI is, therefore, biologically plausible. Our study tested the hypothesis that the development of post-cardiopulmonary bypass AKI was associated with mitochondrial haplogroup. We chose this particular model of AKI because of the easy identifiability and relative homogeneity of the renal insult.

## Results

AKI, as defined by KDIGO (Kidney Disease Improving Global Outcomes) criteria^15^, occurred in 12.8% (113) of the 881 patients retained for final analysis. Demographics and baseline clinical details are shown in Table 1 with a breakdown of their mitochondrial haplogroup profile in Table 2 and clinical outcomes in Table 3. A total of 990 patients had been recruited between 3^rd^ March 2007 and 28^th^ August 2009 with 41 excluded after failing to proceed to surgery (7 died before surgery, in 32 surgery was no longer needed, 2 were withdrawn due to the long gap between recruitment and surgery), 49 excluded after proceeding to off-pump surgery and with 19 excluded due to failed mitochondrial haplotyping.

**Table 1:** Demographics and baseline clinical details of study population (n = 881).

Variable	N	Median (25^th^–75^th^ percentiles)	Range
eGFR (ml/min/1.73 m^2^)	881	66.0 (56.0–76.0)	23–125
<60	290 (32.9%)		
≥60 < 75	342 (38.8%)		
≥75	249 (28.3%)		
BMI (kg/m^2^)	881	29.0 (26.2–32.1)	16–45
<20	10 (1.1%)		
20–25	133 (15.1%)		
>25 </=30	373 (42.3%)		
>30	365 (41.4%)		
Total bypass time (min)	881	78.0 (62.0–97.0)	5–406
≤60	197 (22.4%)		
>60 ≤ 100	489 (55.5%)		
>100	195 (22.1%)		
Number previous heart surgeries	881	0.0 (0- 0)	0–6
≥1	37 (4.2%)		
Age (years)	881	68.0 (61.0–75.0)	20–89
≤60	212 (24.1%)		
>60 < 75	444 (50.4%)		
≥75	225 (25.5%)		
Male gender	613 (69.6%)		
Emergency surgery	51 (5.8%)		
NYHA class 4 (vs. 1–3)	74 (8.4%)		
Hypertensive	575 (65.3%)		
Extra-cardiac arteriopathy present	107 (12.1%)		
COPD^a^ present	77 (8.7%)		
Surgery type			
CABG alone	431 (48.9%)		
Valve alone	287 (32.6%)		
CABG + valve	152 (17.3%)		
Other alone	11 (1.2%)		
Interval from MI^b^ to surgery			
No MI^b^ or MI >30 days prior	848 (96.3%)		
MI 1–30 days prior	33 (3.7%)		
Cerebrovascular disease present	102 (11.6%)		
Left ventricular ejection fraction (%)			
≥30	836 (94.9%)		
<30	44 (5.0%)		
Not measured	1 (0.1%)		
Diabetes management			
Not diabetic	692 (78.5%)		
Diet	33 (3.7%)		
Oral therapy	128 (14.5%)		
Insulin	28 (3.2%)		
Intra-aortic balloon pump (IABP) used	15 (1.7%)		

Median, 25^th^ and 75^th^ percentiles and range are shown for continuous variables* and frequencies for categorical variables.

*Of the continuous variables, only eGFR was normally distributed (Kolmogorov-Smirnov and Shapiro-Wilk test p values > 0.05).

^a^COPD = chronic obstructive pulmonary disease.

^b^MI = myocardial infarction.

**Table 2:** Mitochondrial haplogroup breakdown of study population (n = 881).

Mitochondrial haplogroup	N
HV	411 (46.7%)
J	107 (12.1%)
T	96 (10.9%)
U	127 (14.4%)
K	67 (7.6%)
R	1 (0.1%)
WXI	72 (8.2%)

**Table 3:** Outcome measures of study population (n = 881).

Outcome measure	N	Mean (SD)	Range
KDIGO AKI within 1^st^ 7 days	113 (12.8%)		
Renal support within 1^st^ 7 days	25 (2.8%)		
Maximum AKI stage			
1	85		
2	20		
3 (Cr criteria only)	8		
Hospital mortality	21 (2.4%)		
Hospital length of stay (days)	881	10 (5.6)	1–36

A Chi-square test for homogeneity compared the haplogroup distributions of the study and published control populations^16^ for haplogroups H, U, K, J, T and ‘other’ – the latter comprising those with either low numbers or lack of data in the control population. A significant difference was found (χ^2^ = 11.845, p = 0.037) although, on post hoc analysis using the z test of two proportions with a Bonferroni correction, this seemed to be due to differences in the proportions of haplogroup K (study vs control, 7.6 vs 11.7%) and those in the ‘other’ category (12.3 vs 5%). The frequencies of haplogroup H were comparable (42.7 vs 41.3%).

Chi-square tests for association conducted between KDIGO AKI and the categorical variables shown in Table 4 allowed the identification of potential predictor variables for use in logistic regression analysis (Table 5). These were chosen on the basis of significance (e.g. age, estimated glomerular filtration rate (eGFR) and bypass time categories) or near-significance (e.g. Body Mass Index (BMI) > 30) in the Chi-square analyses or Fisher’s Exact test. The non-binary categorisation of surgery type was chosen over the dichotomous alternative due to the greater clinical credibility of the association.

**Table 4:** Chi-square test for association between KDIGO AKI and the categorical variables, indicated.

Variable (dichotomous ‘yes’ vs. ‘no’, unless otherwise stated)	Pearson Chi-square (χ^2^)	P value	Strength of association (Phi or Cramer’s V **)	P value
Haplogroup* (HV, J, T, U, K, R, WXI)	4.523	0.606		
Gender	1.949	0.163		
Age (≤60 years, >60 < 75, ≥75)	18.736	<0.0005	0.146	<0.0005
Urgency (elective, emergency)	0.442	0.506		
eGFR category (<60 ml/min/1.73 m^2^, ≥60 < 75, ≥75)	40.819	<0.0005	0.215	<0.0005
New York Heart Association (NYHA) class (NYHA 1–3, NYHA 4)	0.294	0.588		
BMI* (<20 kg/m^2^, 20–25, >25–30, >30)	6.082	0.108		
BMI > 30	3.529	0.06		
BMI > 25	0.528	0.468		
Diabetic status	2.752	0.097		
Diabetes management* (not diabetic, diet, oral therapy, insulin)	18.149	<0.0005	0.144	<0.0005
Diabetes management*^a^ (not diabetic or non-insulin-treated, insulin-treated)	18.107	<0.0005	0.143	<0.0005
Hypertension	1.748	0.186		
Extra-cardiac arteriopathy	1.739	0.187		
COPD	0.574	0.449		
Surgery type* (CABG alone, Valve alone, CABG + valve, other surgery alone)	13.543	0.004	0.124	0.004
Surgery type (CABG + valve, single procedure)	4.004	0.045	0.067	0.045
Total bypass time (≤60 mins, >60 ≤ 100, >100)	24.84	<0.0005	0.168	<0.0005
Interval between surgery and MI*^b^ (No MI or MI > 30 days pre-surgery, MI within 30 days pre-surgery)	0.015	0.902		
Cerebrovascular disease	1.522	0.217		
Left ventricular ejection fraction* (≥30%, <30%, not measured)	6.271	0.043	0.084	0.043
Left ventricular ejection fraction (≥30%, <30%) n.b. 1 missing	6.118	0.013	0.083	0.013
Number previous heart surgeries*^c^ (0, ≥1)	4.567	0.033	0.072	0.033
IABP use* (none, pre-operative, intra-operative, post-operative, intra- & post-operative)	25.971	<0.0005	0.172	<0.0005
Any IABP use*^a^	22.393	<0.0005	0.159	<0.0005
H: non-H	0.319	0.572		
J: non-J	0.007	0.932		
T: non-T	0.56	0.454		
U: non-U	0.137	0.711		
K: non-K	0.972	0.324		
HV: non-HV	0.067	0.795		
JT: non-JT	0.238	0.626		
UK: non-UK	0.891	0.345		
WXI: non-WXI	3.071	0.08		

*Failed assumption that all expected cell counts > 5.

**See text for explanation.

^a^Fisher’s exact test significant at p = <0.0005 (2-sided).

^b^Fisher’s exact test non-significant.

^c^Fisher’s exact test significant at p = 0.043 (2-sided).

**Table 5:** Logistic regression predicting likelihood of KDIGO AKI.

Potential predictor variable	Category*	n	B	SE	Wald	df	p	Odds Ratio (OR)	95% C.I. for OR	
									Lower	Upper
Surgery type	CABG alone	431			6.775	3	0.079			
	Valve alone	287	0.173	0.267	0.423	1	0.516	1.189	0.705	2.005
	CABG + valve	152	−0.045	0.328	0.019	1	0.891	0.956	0.503	1.818
	Other alone	11	1.811	0.729	6.179	1	0.013	6.119	1.467	25.524
Age (years)	≤60	212			10.674	2	0.005			
	>60 < 75	444	0.266	0.332	0.640	1	0.424	1.304	0.680	2.501
	≥75	225	0.965	0.358	7.260	1	0.007	2.626	1.301	5.300
Ejection fraction (%)	≥30	836			2.788	2	0.248			
	<30	44	0.700	0.419	2.788	1	0.095	2.014	0.885	4.582
	Not measured	1								
Total bypass time category (mins)	≤60	197			7.591	2	0.022			
	>60 ≤ 100	489	0.375	0.324	1.336	1	0.248	1.455	0.770	2.747
	>100	195	0.956	0.369	6.724	1	0.010	2.603	1.263	5.363
eGFR (ml/min/1.73 m^2^)	≥75	249			16.739	2	<0.0005			
	≥60 < 75	342	0.881	0.370	5.660	1	0.017	2.412	1.168	4.984
	<60	290	1.445	0.367	15.507	1	<0.0005	4.242	2.066	8.707
Intra-aortic balloon pump use	No	866								
	Yes	15	1.203	0.608	3.917	1	0.048	3.329	1.012	10.953
BMI > 30 kg/m^2^	No	516								
	Yes	365	0.394	0.227	3.020	1	0.082	1.483	0.951	2.313
Insulin-treated diabetes	No	853								
	Yes	28	1.433	0.465	9.499	1	0.002	4.190	1.685	10.419
Number of previous heart operations	0	844								
	≥1	37	0.013	0.469	0.001	1	0.977	1.013	0.404	2.542
Haplogroup**	HV	411			4.899	6	0.557			
	WXI	72	0.442	0.357	1.533	1	0.216	1.555	0.773	3.129
	J	107	−0.111	0.352	0.100	1	0.752	0.895	0.449	1.782
	T	96	−0.040	0.387	0.011	1	0.918	0.961	0.450	2.052
	U	127	−0.222	0.339	0.431	1	0.512	0.801	0.412	1.556
	K	67	−0.718	0.494	2.111	1	0.146	0.488	0.185	1.285
Constant			−4.156	0.493	71.137	1	<0.0005	0.016		

Forty-two studentized residuals with values > 2.5 standard deviations (range 2.582–6.079) were all retained.

*The first category for each potential predictor variable represents the reference category (e.g. “CABG alone” for “Surgery type”).

**The study cohort also included a single case with haplogroup ‘R’ which did not contribute, significantly, to the model.

The logistic regression model (Table 5) was statistically significant (χ^2^ = 95.483, p < 0.0005), explained 19.2% of the variance in KDIGO AKI (Nagelkerke R^2^) and correctly classified 87.7% of cases. As shown in Table 5, six of the ten predictor variables were statistically significant: age, eGFR, the presence of insulin-treated diabetes, bypass time, balloon pump use and ‘other’ cardiac bypass surgeries (non-coronary artery bypass graft (CABG), non-valve procedures although numbers were small and 95% confidence intervals were wide). Neither the phylogenetic supergroups, WXI and HV, nor any individual haplogroup was a significant predictor of KDIGO AKI in this model.

Supplementary Chi-square tests for association showed no significant associations between higher stages of AKI and individual haplogroups or the phylogenetic groupings, WXI or HV, although numbers of cases were small (as shown in Table 3).

Chi-square tests for association undertaken between each of the 6 predictor variables identified above and haplogroup failed to reveal any significant association although at least 3 breaches of cell frequency assumptions were evident in each test.

In view of the failure of cell frequency assumptions in the Chi-square tests for association, noted above, supplementary analyses were undertaken. Firstly, haplogroup:non-haplogroup variables were created for the common mutations within the dataset (e.g. H:non-H, etc.) and for phylogenetic supergroups, which also incorporated less common mutations (e.g. WXI:non-WXI) – an approach in keeping with our previous published work^13^. Each pairing was then utilised in Chi-square tests for association against KDIGO AKI. As shown in Table 4, none were significant but all met cell frequency assumptions. The WXI:non-WXI pairing was then substituted for the multinomial haplogroup variable used in our initial logistic regression model which remained statistically significant (χ^2^ = 92.695, p < 0.0005), now explained 18.7% of the variance in KDIGO AKI (Nagelkerke R^2^) and correctly classified 87.9% of cases. Regression analyses were repeated, sequentially, substituting each haplogroup:non-haplogroup pairing into the model. No pairing was a significant predictor of KDIGO AKI when introduced into this model.

Chi-square tests for association undertaken between each of the 6 predictor variables identified above and each of the 9 haplogroup:non-haplogroup pairings revealed significant associations between T:non-T and eGFR (χ^2^ = 8.425 (p = 0.015; Cramer’s V < 0.1)), and between HV:non-HV and both bypass time (7.783 (0.020; Cramer’s V < 0.1)) and balloon pump use (4.365 (0.037; Phi −0.07)) although the strength of these associations was weak. In view of the multiple hypotheses under test (n = 54), we applied a Bonferroni correction at a significance level of α (0.05)/κ (54) and the Benjamini-Hochberg technique^17^, neither of which found any of these associations to remain significant.

Because of a biologically plausible association between haplogroup and the risk of chronic kidney disease (CKD), additional logistic regression analysis allowed further observations on T:non-T and eGFR. To facilitate this, eGFR was re-categorised as a binomial variable. This was undertaken at two, separate thresholds, chosen to map to the limits of the original multinomial function (Table 1): (1) eGFR < 60 vs ≥ 60, and (2) eGFR < 75 vs ≥ 75 (‘eGFR 75’). The association between T:non-T and eGFR 75 was significant (χ^2^ 8.120, p = 0.004; Phi 0.096). Logistic regression analysis, employing eGFR 75 as the dependent variable, identified the following significant predictor variables: the T:non-T pairing, age, gender and New York Heart Association (NYHA) class. The model was statistically significant, χ^2^ = 96.342, p < 0.0005, with non-T haplogroup observed as conferring a 1.86 times higher odds of a baseline eGFR < 75 (95% CI 1.162–2.973, p = 0.010).

## Discussion

Human mitochondrial DNA (mtDNA) encodes 13 protein components of the mitochondrial respiratory chain^18^. Its matrilineal inheritance has led to the accumulation of specific single nucleotide polymorphisms (SNPs) which have allowed the mitochondrial genome to be characterized according to different mitochondrial haplogroups^19^. These haplotypes may well be of functional significance^9–13^ although an association between mitochondrial haplogroup and susceptibility to AKI has yet, to our knowledge, to be described. Nevertheless, it is clear that mitochondria play a critical role in both tissue injury and repair in AKI^7^.

Structural abnormalities such as swelling of individual mitochondria, depletion in total numbers and disruption of cristal architecture have been noted from the early stages of disease, particularly in the proximal tubule, and appear to accompany a marked reduction in respiratory chain activity^7^. As well as deficits in adenosine triphosphate production, mitochondrial disruption leads to the accumulation and release of molecules that can propagate inflammation, cell injury and apoptosis; these include non-esterified free fatty acids, reactive oxygen species, cytochrome c and mtDNA, itself^7^. Conversely, their importance in recovery from AKI is evidenced by both increases in mitochondrial biogenesis and the negative impact of reduced mitophagy on renal recovery^7^. Therapeutic manipulation of both injury and recovery processes is now being explored and is discussed in more detail in reference^7^. Interestingly, mitochondrial dysfunction may be important in increasing even the susceptibility to AKI with experimental *in vitro* and *in vivo* work describing the ‘priming’ of mitochondria to apoptosis in hyperglycaemic environments^20^.

Beyond AKI, both inherited and acquired mitochondrial abnormalities have been described in a range of other renal diseases. These often occur as part of multi-system disorder in children and young adults and are frequently clinically silent^7^. Disorders of tubular function predominate, as might be expected given the high local energy demands, but mitochondrial dysfunction is also evident in different cystic and glomerular diseases (see references in^7^).

A number of SNPs in the mitochondrial and nuclear genomes affecting mitochondrial function have also been associated with more prevalent renal diseases such as type 1^21^ and type 2 diabetic nephropathy^22^, with reduced kidney survival in CKD^23^ and as predictors of chronic dialysis^24^. The common, acquired mtDNA^4977^ deletion mutation was found to be associated with improved survival in a patient cohort from the HEMO study and, although the explanation for this preliminary observation was not clear, there was a trend for these patients to have higher mtDNA copy number^25^. The potential importance of epigenetic factors, has been illustrated by an association between type 1 diabetic kidney disease and the DNA methylation of nuclear genes influencing mitochondrial function^26^.

Beyond these genetic factors, mitochondrial dysfunction appears to increase with worsening CKD (see references in^27^) and seems to have a central role in the development of the oxidative stress associated with deteriorating renal function (see references in^28^).

To our knowledge, the present study is the first examination of the hypothesis that mitochondrial haplogroup influences the risk of developing AKI. The supposition carries biological plausibility given the pivotal role of mitochondria in pathogenesis and because subtle differences of function across haplogroups may confer differing levels of risk^8^. We found no influence on the risk of post-cardiac bypass AKI or, in supplementary analysis, on the severity of AKI. Our negative findings should be viewed in the context that this was a single-centre study examining a specific aetiology of AKI.

We also note that, although the main logistical regression model was sufficiently powered for the number of potential predictors (Table 5) and AKI incidence (see ‘Statistical analysis’, below), a more subtle effect of haplogroup may have been detected in a larger cohort. This should be placed in the context of ours being one of the larger cohorts with this phenotype, though.

Our primary analysis utilised the ‘haplogroup’ variable, comprising the different haplogroups and phylogenetic supergroups as described under ‘Statistical analysis’, below, and elsewhere^29^. This multinomial categorisation did lead to a failure of cell frequency assumptions in the Chi-square tests for association although this was entirely due to inclusion of the single case with ‘R’ haplotyping. Our supplementary analysis, substituting the ‘haplogroup’ variable with ‘haplogroup:non-haplogroup’ was conducted with the aim of increasing the denominator (by creating the ‘non-haplogroup’ category) – a technique used, extensively, including in previous published work from our group^13^. However, this approach assumes an independence between categories that may not be correct as the ‘non-haplogroup’ category may contain states that have evolutionary proximity to the ‘haplogroup’ state of the pairing (e.g. ‘non-J’ includes ‘T’, with both ‘J’ and ‘T’ classifiable in the same phylogenetic supergroup, ‘JT’; similarly ‘non-U’ including ‘K’ and ‘non-T’ including ‘J’).

We believe, however, that the approach in this supplementary analysis is justified as a means of further exploring potential associations of interest and note, also, that the multinomial categorisation used for the primary ‘haplogroup’ variable (HV, J, T, U, K, R, WXI) does not fully resolve the issue of association between different states (‘J’ and ‘T’, ‘U’ and ‘K’, etc.).

Given the major role of mitochondrial dysfunction in AKI, our findings require replication in other patient groups, including in other types of AKI, and should extend to other abnormalities of mitochondrial and related nuclear genetics beyond, simply, haplotype.

Our finding of an association between mitochondrial haplogroup T (versus ‘non-T’) and CKD was unexpected and could only be regarded as anecdotal in the context of multiple hypothesis testing. In contrast to AKI, however, inherited differences in the mitochondrial genome have, indeed, been associated with CKD.

For instance, haplogroup D was found to be a risk marker for end-stage renal disease in young, Han Chinese subjects^30^. Further, mtDNA haplogroups J and V have been associated with reduced susceptibility to chronic renal allograft dysfunction in comparison to haplogroup H^31^ which has also been associated with a higher risk of the development of post-renal transplant diabetes^32^. Finally, a gene-environment interaction study suggested an association between the longevity-associated mt5178A genotype, regular alcohol consumption and better renal function in non-diabetic Japanese men^33^.

In conclusion, the present study has found no significant association between common European haplogroups and the risk of AKI after cardiac bypass surgery. Our observations require replication in other patient cohorts and with different aetiologies of AKI.

## Methods

Ethical approval for the study protocol and procedures was received from the Northumberland Local Research Ethics Committee (ref: 07/Q0902/30) in accordance with their ethical standards and with the Helsinki Declaration of 1975, as revised in 2000.

Consecutive patients were screened for inclusion from the institution’s adult cardiac surgery pre-assessment clinic and in-patient wards prior to elective or emergency cardiac bypass surgery. Exclusion criteria comprised: age <18 years, pre-existing chronic dialysis or renal transplant, stage 5 CKD (eGFR < 15 mL/min/1.73 m^2^), pre-existing AKI (defined by contemporary consensus criteria^34^), patients undergoing cardiac or lung transplantation, participation in other studies and non-Caucasian race. After signed, written informed consent, a 10 mL EDTA blood sample was collected from each recruited patient. Genomic DNA was extracted (“Nucleon® Total Genomic DNA Extraction Kit:” product 44100, Tepnel Life Sciences PLC) and mitochondrial DNA haplotype analysis performed using polymerase chain reaction and restriction fragment polymorphism analysis as previously described^35,36^.

Data extracted from the clinical record included demographics, pre-operative co-morbidity, surgery type, the presence of recognised risk factors for post-cardiac bypass AKI^2–5,37–39^ and outcome measures (the need for renal replacement therapy (RRT) within the 1^st^ 7 post-operative days, hospital length of stay, and in-hospital mortality). Post-bypass AKI was determined using the baseline of the pre-operative serum creatinine (SCr) from the day prior to surgery or sooner or, if unavailable, the pre-assessment clinic SCr. KDIGO (Kidney Disease Improving Global Outcomes) criteria^15^ were utilised in our analyses to diagnose AKI and stage its severity over the 7 days post-surgery. Patients listed for cardiac bypass surgery but subsequently undergoing an off-pump procedure were excluded from further analysis because of the possible impact on AKI risk^1^.

SCr was measured using the Jaffé method on an Olympus AU Analyser (Beckman Coulter (UK) Ltd, High Wycombe). In order to determine baseline eGFR, the corresponding SCr was adjusted to harmonise with gold standard methodology (Isotope Dilution Mass Spectrometry) using the following correction factor, provided by the UK-accredited Wales External Quality Assessment Scheme^40^ in 2006:

$$({\rm{SCr}}-11)\times 1.04$$

Baseline eGFR was then calculated using the revised four-variable MDRD 175 formula for SI units^41^.

### Statistical analysis

An independent, multinomial variable, ‘haplogroup’ was created comprising the following categories: J, T, U, K, WXI, R and HV, with the latter used as reference.

The primary, dichotomous dependent variable was post-operative AKI (‘KDIGO AKI’) defined solely by SCr criteria^15^, namely: a rise from baseline of ≥26.5 μmol/L within 48 hours or ≥1.5 fold within 7 days.

A Chi-square test for homogeneity^42^ compared the haplogroup distributions of the study population and a previously described local control population^16^.

Chi-square tests for association^43^ were then undertaken between KDIGO AKI and each potential predictor variable, including the multinomial haplogroup variable. Continuous variables (e.g. age, eGFR) were converted to categorical variables for these analyses. Tests were conducted under the assumption that expected cell frequencies were >5 but where this failed, Fisher’s Exact test was undertaken if the tested variable was dichotomous. The strength of any significant association was tested using the Phi statistic for dichotomous variables and Cramer’s V, otherwise, with both measures interpreted in the same manner as a correlation (i.e. ranging from −1 to +1 and from 0 to +1, respectively). Sensitivity analyses, involving re-categorisation of other variables with potentially significant associations, allowed identification of additional potential predictors for use in the regression analysis.

Supplementary Chi-square tests for association were performed to evaluate the association of the multinomial haplogroup variable with stage 2 and/or stage 3 AKI.

Binomial logistic regression analysis was then performed^44^ using KDIGO AKI as the dependent variable and, as independent variables, the multinomial haplogroup variable and any other independent variables, found to be significant in the Chi-square tests for association, described above.

The impact of haplogroup on any other significant predictors identified from the regression model was then explored with further Chi-square tests for association (predictor variable vs haplogroup) and binomial logistic regression (if the association was significant and clinically credible).

Supplementary analyses were then performed.

Firstly, haplogroup:non-haplogroup variables were created for the common mutations within the dataset (e.g. H:non-H, etc.) and for phylogenetic supergroups, which also incorporated less common mutations (e.g. WXI:non-WXI). Chi-square tests for association^43^ were then undertaken between KDIGO AKI and each haplogroup:non-haplogroup pairing. Each binomial pairing was then, sequentially, substituted for the multinomial ‘haplogroup’ variable used in the primary logistic regression model for prediction of KDIGO AKI. Finally, the impact of each haplogroup:non-haplogroup pairing on other, significant predictors of KDIGO AKI, identified from this regression model was then explored with further Chi-square tests for association (predictor variable vs haplogroup:non-haplogroup pairing) and binomial logistic regression (if the association was significant and clinically credible).

Following the recommendation that there should be at least 10 events per variable studied^45^, for a 10 variable model, 100 events would have to occur in the study population; thus, for the reported AKI incidence of 18%^1^, target recruitment would be 556 whilst lower incidences of 15, 12.5 and 10% would increase target recruitment to 667, 800 and 1000 patients, respectively.

Analyses were performed using the *SPSS statistics* package (*version 22, IBM Corporation*) with p < 0.05 being regarded as statistically significant. Where percentages are reported (e.g. Tables 1–3), these are rounded to one decimal place.

## Data Availability

The datasets generated during and analysed during the current study are available from the corresponding author on reasonable request.
